# A regimen based on the combination of trimethoprim/sulfamethoxazole with caspofungin and corticosteroids as a first-line therapy for patients with severe non-HIV-related pneumocystis jirovecii pneumonia: a retrospective study in a tertiary hospital

**DOI:** 10.1186/s12879-024-09031-7

**Published:** 2024-01-31

**Authors:** Hao Li, Yihe Lu, Guoxin Tian, Yongxing Wu, Tianjun Chen, Jiangwei Zhang, Nan Hu, Xiaoning Wang, Yang Wang, Lan Gao, Jinqi Yan, Linjing Zhou, Qindong Shi

**Affiliations:** 1 Department of Critical Care Medicine, the First Affiliated Hospital of Xi’an Jiaotong University, 277 Yanta West Road, Xi’an, Shaanxi China; 2 Shaanxi Provincial Key Laboratory of Sepsis in Critical Care Medicine, Xi’an, Shaanxi China; 3 Department of Respiratory and Critical Care Medicine, the First Affiliated Hospital of Xi’an Jiaotong University, 277 Yanta West Road, Xi’an, Shaanxi China; 4 Department of Kideny Transplant, Hospital of Nephrology, the First Affiliated Hospital of Xi’an Jiaotong University, 277 Yanta West Road, Xi’an, Shaanxi China; 5 Department of Rheumatology, the First Affiliated Hospital of Xi’an Jiaotong University, 277 Yanta West Road, Xi’an, Shaanxi China; 6 Department of Hematology, the First Affiliated Hospital of Xi’an Jiaotong University, 277 Yanta West Road, Xi’an, Shaanxi China; 7 Department of Cardiovascular Medicine, the First Affiliated Hospital of Xi’an Jiaotong University, 277 Yanta West Road, Xi’an, Shaanxi China

**Keywords:** Pneumocystis Jirovecii pneumonia, Clinical response, Adverse event, Sulfamethoxazole-trimethoprim, Caspofungin, Glucocorticosteroid

## Abstract

**Background:**

Pneumocystis jirovecii pneumonia (PJP) is a life-threatening and severe disease in immunocompromised hosts. A synergistic regimen based on the combination of sulfamethoxazole-trimethoprim (SMX-TMP) with caspofungin and glucocorticosteroids (GCSs) may be a potential first-line therapy for PJP. Therefore, it is important to explore the efficacy and safety of this synergistic therapy for treating non-HIV-related PJP patients.

**Methods:**

We retrospectively analysed the data of 38 patients with non-HIV-related PJP at the First Affiliated Hospital of Xi’an Jiaotong University. Patients were divided into two groups: the synergistic therapy group (ST group, *n* = 20) and the monotherapy group (MT group, *n* = 18). All patients were from the ICU and were diagnosed with severe PJP. In the ST group, all patients were treated with SMX-TMP (TMP 15–20 mg/kg per day) combined with caspofungin (70 mg as the loading dose and 50 mg/day as the maintenance dose) and a GCS (methylprednisolone 40–80 mg/day). Patients in the MT group were treated only with SMX-TMP (TMP 15–20 mg/kg per day). The clinical response, adverse events and mortality were compared between the two groups.

**Results:**

The percentage of patients with a positive clinical response in the ST group was significantly greater than that in the MT group (100.00% vs. 66.70%, *P* = 0.005). The incidence of adverse events in the MT group was greater than that in the ST group (50.00% vs. 15.00%, *P* = 0.022). Furthermore, the dose of TMP and duration of fever in the ST group were markedly lower than those in the MT group (15.71 mg/kg/day vs. 18.35 mg/kg/day (*P* = 0.001) and 7.00 days vs. 11.50 days (*P* = 0.029), respectively). However, there were no significant differences in all-cause mortality or duration of hospital stay between the MT group and the ST group.

**Conclusions:**

Compared with SMZ/TMP monotherapy, synergistic therapy (SMZ-TMP combined with caspofungin and a GCS) for the treatment of non-HIV-related PJP can increase the clinical response rate, decrease the incidence of adverse events and shorten the duration of fever. These results indicate that synergistic therapy is effective and safe for treating severe non-HIV-related PJP.

## Introduction

Pneumocystis jirovecii pneumonia (PJP) is a life-threatening and severe disease in immunocompromised hosts [1]. Compared with HIV-positive PJP patients, non HIV-positive PJP patients tend to develop acute respiratory failure, which requires admission to the intensive care unit (ICU) [2]. Furthermore, the mortality rate among non-HIV-related PJP patients is higher than that among HIV-positive PJP patients, which ranges from 34 to 58%, despite the utilization of the first-line antimicrobial agent sulfamethoxazole-trimethoprim (SMX-TMP) [3]. On the other hand, multiple adverse effects, such as thrombocytopenia, hyperkalaemia and diarrhoea, are associated with SMX-TMP, which limits its utilization. Therefore, it is important to explore effective antimicrobial regimens for treating non-HIV-related PJP patients and decreasing adverse effects.

Based on the ECIL guidelines and limited case reports, in addition to the first-line agent SMX-TMP, the combination of SMX-TMP with caspofungin may exhibit synergistic antimicrobial effects against *Pneumocystis Jirovecii* and may constitute an alternative treatment regimen for patients with non-HIV-related PJP [4, 5]. Furthermore, a pilot study in mice conducted by Lobo et al. demonstrated that the administration of caspofungin in combination with SMX-TMP may provide an improved treatment protocol for *Pneumocystis Jirovecii* infection clearance [6]. However, previous studies have shown that caspofungin combined with SMX-TMP as a first-line therapy for non-HIV-related PJP is very limited, and the results are inconsistent. Kim et al. indicated that 4 non-HIV-related PJP patients who received caspofungin combined with SMX-TMP as a therapeutic regimen showed no response [7]. In contrast, the study by Zhang et al., which included a small sample of 14 patients, showed that caspofungin combined with SMX-TMP may be an effective strategy for treating non-HIV patients with severe PJP [8].

On the other hand, Pareja et al.’s study based on a small sample indicated that adjunctive glucocorticosteroids (GCSs) may accelerate recovery in adult patients with severe non-HIV-related PJP [3]. However, the routine adjunctive use of GCSs in non-HIV-related PJP patients is not recommended in the ECIL guidelines, and the dosage and duration of GCSs for treating non-HIV-related PJP are unclear; moreover, additional evidence is needed to determine the benefit of utilizing GCSs.

Therefore, we performed this study to determine whether a synergistic regimen based on the combination of SMX-TMP with caspofungin and GCSs as a first-line therapy could exert an effective antimicrobial effect and decrease the incidence of adverse effects when treating severe non-HIV-related PJP patients.

## Materials and methods

### Patient enrolment and data collection

This retrospective observational study was performed from October 2018 to September 2022 at the First Affiliated Hospital of Xi’an Jiaotong University.

The study was approved by the Ethics Review Board of the First Affiliated Hospital of Xi’an Jiaotong University (No. XJTU1AF2019LSL-016). Patients who were diagnosed with severe PJP and admitted to the ICU were eligible for inclusion. Severe PJP was defined as hypoxemic acute respiratory failure requiring high-flow nasal oxygen with at least 50% FiO_2_, noninvasive ventilation, or mechanical ventilation (MV) [8–10]. Patients who did not meet the criteria for severe PJP were considered to have mild-to-moderate PJP, and the classification criteria were as follows: mild, PaO2 > 70 mmHg while breathing room air; and moderate, PaO2 ≤ 70 and PaO2 > 60 mmHg [8–10]. The exclusion criteria were as follows: (i) < 18 years of age, (ii) pregnant, (iii) HIV-positive, and (iv) mild-to-moderate PJP. A PJP diagnosis was defined as the presence of findings indicating possible PJP detected by a radiologist via chest computed tomography combined with positive results on next-generation sequencing (NGS) of sputum or bronchoalveolar lavage fluid (BALF). Patients who received a synergistic regimen as first-line therapy were identified as the ST group. The synergistic regimen based on the combination of SMX-TMP with caspofungin and a GCS to treat non-HIV-related PJP was as follows: SMX-TMP (TMP 15–20 mg/kg per day for at least 21 days) combined with caspofungin (70 mg as the loading dose and 50 mg/day as the maintenance dose) and a GCS (methylprednisolone 40–80 mg/day for 3–14 days, after which the dose was decreased to the baseline dosage or treatment was stopped). Patients who received only SMX-TMP (TMP 15–20 mg/kg per day) as a first-line monotherapy were defined as the MT group. The medical information of all the patients, including baseline demographic data, clinical scores, outcomes, and laboratory test results, was collected and carefully recorded. These anonymized clinical data were collected from the Biobank of the First Affiliated Hospital of Xi’an Jiaotong University.

### Serum β-(1,3)-D-glucan test

The serum β-(1,3)-D-glucan (BG) concentration was measured via the Fungus(1–3)-β-D-Glucan Test (Gold Mountainriver Co., Beijing, China). If the test value was higher than 100 pg/mL, fungal infection was highly suspected.

### Renal replacement therapy

Continuous veno-venous haemofiltration (CVVH) was performed by using Prismaflex (Gambro Co., Ltd.) or multiFiltrate (Fresenius Medical Care GmbH, Bad Homburg, Germany) machines. The anticoagulation regimen consisted of low-molecular-weight heparin or citrate. The blood flow rate was set at 120–200 ml/min.

### Extracorporeal membrane oxygenation

Veno-venous extracorporeal membrane oxygenation (VV-ECMO) was established by placing 17 and 21 F cannulas into the right intrajugular vein and right femoral vein, respectively, under sterile conditions. During cannulation, 100 units/kg heparin was administered to the patient, and the whole-blood activated clotting time (ACT) was measured every 2–4 h to maintain an ACT at 160–180 s [11]. Extracorporeal bypass was initiated with a blood flow of 3.0–4.0 L/min and a sweep gas of 4.0–5.0 L/min. During ECMO, the ventilator setting followed a protective ventilatory strategy to minimize the risk of ventilator-induced lung injury [12].

### Method of bronchoscopy and sputum induction

To identify the *Pneumocystis jirovecii* pathogen, next-generation metagenomics sequencing (mNGS) of bronchoalveolar lavage fluid (BALF) or sputum was performed. After informed consent was obtained, bronchoscopy was performed via the Olympus BF-Q170 electronic bronchoscope (the outer diameter of the insertion part was 4.8 mm, and the working channel was 2.2 mm) transnasally or through an endotracheal tube. Then, bronchoalveolar lavage fluid (BALF) was acquired in accordance with standardized operating procedures for further mNGS [13].

Sputum induction was performed with 5% saline solution as previously described, after which samples were collected by expectoration or, in patients who could not expectorate, by nasopharyngeal suction or oropharyngeal suction in those who refused suctioning or were uncooperative with the procedure [14]. To determine the quality of the sample, sputum was examined in accordance with standard procedures by a microbiologist to determine whether the sample was representative of lower respiratory tract secretions [15].

### mNGS

All BALF and sputum samples were analysed via mNGS. The specimen preparation, bacteriological procedures, sequencing methods, and metagenomic data analysis were performed via standardized operating procedures [16, 17]. Generally, mNGS results are available within 48 h.

### Clinical response assessment

A clinical response to PJP treatment was defined as a positive response or failed response. A positive response was defined as follows: (i) amelioration or resolution of baseline signs, symptoms (fever, cough, dyspnoea, respiratory rate and pulse rate) or chest roentgenographic findings and (ii) decreased oxygen requirements following therapy [7, 8]. Treatment failure was defined as follows: (i) progressive clinical deterioration, as demonstrated by the inability to maintain a stable partial pressure of arterial oxygen despite an increase in the fraction of arterial oxygen; and (ii) progressive deterioration of vital signs with a requirement for an increased fraction of inspired oxygen after 7 days of therapy [7, 8].

### Definition of SMX-TMP-associated adverse events

Data on SMX-TMP-associated adverse events were recorded. As previously described, the side effects of SMX-TMP include thrombocytopenia, hypoglycaemia, photosensitivity, hyperkalaemia, diarrhoea, hepatotoxicity and aplastic anaemia [18]. All SMX-TMP-associated side effects are reversible after treatment withdrawal. Considering that the enrolled patients were from the ICU and had a high incidence of organ dysfunction, all reported cases of adverse drug events were reanalysed by a clinical pharmacist.

### Statistical analysis

The statistical analysis was performed with SPSS version 16.0 (SPSS, Inc., Chicago, IL, USA). Categorical data are presented as proportions, and continuous data are presented as medians (interquartile ranges [IQRs]). Independent t tests were used to compare continuous variables between the two groups with a normal distribution. Nonnormally distributed continuous variables and differences between the two groups were analysed by using the Mann‒Whitney U test. *P* < 0.05 was considered to indicate statistical significance.

## Results

### Patient characteristics

From October 2018 to September 2022, 38 patients (25 males and 13 females) were enrolled in the present study—20 patients were included in the ST group and 18 patients were included in the MT group. As shown in Table 1, there were no significant differences in baseline parameters between the MT group and the ST group. Since the population we studied was immunocompromised, multiple pathogens could be detected in a single patient’s sample, and the coinfection rate was high. Pathogens were detected in a total of 26 samples (68.42%, 26/38), including 13 samples with bacteria (36.84%, 14/38), 11 samples with viruses (34.21%, 11/38) and 2 samples (5.26%, 2/38) with fungi. As shown in Table 2, the most common underlying disease was renal transplantation (50.00%, 19/38). Table 3 lists the characteristics of the 20 patients in the ST group. Three patients (25.00%, 4/20) in the ST group underwent extracorporeal membrane oxygenation (ECMO). The dosage and duration of methylprednisolone were 0.96 ± 0.46 mg/kg/day and 6.71 ± 2.57 days, respectively. Since most of the patients in the ST group were administered long-term prednisone to control their underlying diseases, we analysed only the duration and dosage of methylprednisolone. On the other hand, the mean duration of caspofungin use was 20.65 ± 8.94 days.

**Table 1:** Sociodemographic characteristics of patients

Parameters	Total	ST group	MT group	*P*
No. of patients	38	20	18	
Age, yr [median (IQR)]	59.50(37.75-62.00)	54.00(40.25-62)	46.00(36.50-56.75)	0.273
Gender, male/female	25/13	13/7	12/6	0.915
Bodyweight, kg, [median (IQR)]	61.00(55.00-67.25)	62.00(55.00-68.75)	59.50(55.75–65.5)	0.568
PaO_2_/FiO_2_	199.85(114.44-238.89)	181.75(83.63-225.88)	224.35(163.75-240.11)	0.066
Scores				
APACHE II score, [median (IQR)]	19.00 (13.00-25.25)	19.00(17.00-25.75)	19.50(9.00-25.50)	0.235
SOFA score, [median (IQR)]	7.00(5.00-9.25)	7.00(6.00–10.00)	6.00(2.00–8.00)	0.086
Vasopressor utilization ratio,[%. no.]	37.10 (13/35)	45.00(9/20)	27.80(5/13)	0.278
CRRT utilization ratio, [%. no.]	40.00(14/35)	40.00(8/20)	44.40(8/10)	0.785
Lab test				
Albumin, g/L	27.20(23.55–31.60)	28.05(24.52–31.83)	24.75(21.68–28.95)	0.111
LDH, U/L, [median (IQR)]	600.50(367.25–725.00)	629.50(391.50-807.25)	512.50(326.25–725.00)	0.492
C-reactive protein, mg/L, [median (IQR)]	64.68(33.95-135.28)	71.70(54.60-162.73)	49.70(22.15–91.05)	0.068
Interleukin-6, pg/mL, [median (IQR)]	77.74(23.40-311.53)	59.88(22.74-359.98)	112.15(28.65-309.83)	0.792
Procalcitonin, ng/mL, [median (IQR)]6	1.13(0.44–3.43)	1.15(0.48–5.69)	0.81(0.32–2.01)	0.306
CD_4_ count, cells/µL, [median (IQR)]	124.50 (85.21-171.75)	98.99(68.50-148.75)	142.50(113.00-184.25)	0.059
Serum β-(1,3)-D-glucan, pg/mL, [median (IQR)]	197.14(120.34-271.53)	207.7(121.95-319.79)	184.42(111.60-267.78)	0.693
Total Other pathogen dectected, [%. no.]	68.42(26/38)	75.00(15/20)	61.11(11/18)	0.364
Bacteria	36.84(14/38)	40.00(8/20)	33.33(6/18)	
Virus	34.21(13/38)	45.00(9/20)	22.22(4/18)	
Fungus	5.26(2/38)	5.00(1/20)	5.56(1/18)	

ST group, synergic therapy group; MT group, monotherapy group; APACHE II score, acute Physiology and Chronic Health Evaluation II score; SOFA score, sepsis related Organ Failure Assessment score; CRRT, continuous renal replacement therapy; LDH, lactate dehydrogenase; PEEP, positive end expiratory pressure; Vt, tidal volume; PBW, predicted body weight

**Table 2:** Admission unit and underlying diseases of patient

Underlying diseases	Total, *n* = 38	ST group, *n* = 20	MT group, *n* = 18
Renal transplantation, [%. no.]	50.00 (19)	50.00(10)	50.00(9)
Haematological malignancies, [%. no.]	15.79(6)	20.00(4)	11.11(2)
Nephropathy, [%. no.]	7.89(3)	5.00(1)	11.11(2)
Vasculitis, [%. no.]	7.89(3)	5.00(1)	11.11(2)
Lupus erythematosus, [%. no.]	7.89(3)	10.00(2)	5.56(1)
Polymyositis, [%. no.]	5.26(2)	5.00(1)	5.56(1)
Mediastinal sacroma, [%. no.]	2.63(1)	0.00(0)	5.56(1)
Herpes zoster, [%. no.]	2.63(1)	5.00(1)	0.00(0)

ST group, synergic therapy group; MT group, monotherapy group

**Table 3:** Clinical characteristics of the cases in ST group

Case	Age, year	Gender	Underlying condition	Tracheal intubation	ECMO	CRRT	Co-infection/Location	Duration of Caspo, day	MP, mg/kg/day	Duration of MP, day	Outcome
1	59	M	Renal transplantation	Y	N	N	CMV/Lung	17	1.16	7	Survived
2	59	F	Nephropathy	Y	N	N	KP/Lung	22	1.51	7	Survived
3	62	M	acute myeloid leukemia	Y	N	Y	PA/Lung	21	1.36	5	Survived
4	62	M	Vasculitis	Y	N	N	*C. albicans /Lung*	15	1.23	8	Survived
5	76	M	Polymyositis	Y	N	N	MRSA /Lung	24	0.62	5	Survived
6	51	M	Renal transplantation	N	N	Y	*E. coli*/urinary tract	16	1.67	3	Survived
7	25	M	Lupus erythematosus	Y	N	N		27	1.54	5	Survived
8	36	M	Renal transplantation	Y	Y	N	CMV/Lung	23	0.65	5	Survived
9	79	M	Herpes zoster	Y	N	Y	CRAB/Lung and blood	31	1.07	7	Died
10	34	M	Renal transplantation	N	N	Y		25	0.53	7	Survived
11	54	M	acute myeloid leukemia	Y	N	Y	CMV/Lung	12	0.58	5	Survived
12	65	M	acute myeloid leukemia	Y	Y	N	CRAB/Lung	31	0.65	14	Survived
13	64	F	Lupus erythematosus	Y	Y	Y	HPV type IV /Lung + EF/Blood	36	0.42	5	Survived
14	50	F	Renal transplantation	Y	N	N	HPV type I I/Lung	9	1.60	9	Died
15	54	M	acute myeloid leukemia	Y	N	N		5	0.66	5	Died
16	47	M	Renal transplantation	N	N	N	CMV + HPV type IV/Lung	8	0.24	7	Survived
17	49	F	Renal transplantation	N	N	N	CMV/Lung	29	0.77	7	Survived
18	19	F	Renal transplantation	N	N	N	SARS-CoV-2/Lung	7	0.40	5	Survived
19	56	F	Renal transplantation	Y	Y	Y	MRSA/Lung	31	0.72	10	Died
20	38	F	Renal transplantation	Y	N	Y	CMV + EBV + VZV/Lung	6	0.73	3	Died

Caspo, Caspofungin; CMV, *Cytomegalovirus*; VZV, *Human alphaherpesvirus*; EBV, *Human gammaherpesvirus 4*; HPV, *Human herpesvirus*; KP, *Klebsiella pneumoniae*; PA, *Pseudomonas aeruginosa*; *C. albicans*, *Candida albicans*; MRSA, Methicillin-resistant *Staphylococcus aureus*; EF, *Enterococcus faecium*; *E. coli*, *Escherichia coli*; SARS-CoV-2, severe acute respiratory syndrome coronavirus 2; CRAB, Carbapenem Resistance *Acinetobacter Baumannii*; MP, methylprednisolone

### Clinical response and adverse effects of SMZ-TMP

The clinical response was evaluated, and a total of 32 patients were documented to have a positive response to PJP treatment (Table 4). The percentage of patients with a positive clinical response in the ST group was 100.00%, which was significantly greater than that in the MT group (*P* < 0.05). Treatment failure was reported in 6 patients (33.33%, 6/18) in the MT group, and all 6 of these patients received salvage therapy. Finally, 3 patients were successfully treated with salvage synergistic therapy, 2 patients died after treatment failure, and 1 patient refused invasive mechanical ventilation, left the ICU and ultimately died the next day. There were no significant differences in all-cause mortality or duration of hospital stay between the MT group and the ST group. After successful treatment of *P. jirovecii* infection, five patients in the ST group died. One patient developed acute kidney failure, refused renal replacement therapy and ultimately died of electrolyte disturbance. One patient died after cessation of ECMO, and another died of severe infection caused by multiresistant *Acinetobacter baumannii*. Two patients refused ECMO and died of respiratory failure (Table 3). On the other hand, the incidence of all adverse events and the dose of TMP in the ST group were significantly lower than those in the MT group (*P* < 0.05), and the duration of fever was markedly shorter in the ST group than in the MT group (*P* < 0.05).

**Table 4:** Clinical response and associated adverse events

	Total, *n* = 38	ST group, *n* = 20	MT group, *n* = 18	*P*
Positive of clinical response [%, no.]	84.21(32/38)	100.00(20/20)	66.70(12/18)	0.026^*^
Duration of hospital stay	20.50(10.75–31.25)	30.00(10.50–42.50)	15.00(9.00-22.25)	0.059
Duration of fever	8.00(6.00-10.25)	7.00(5.00-8.75)	11.50(8.00-16.25)	0.029^*^
Dose of TMP, mg/kg/day	16.55(15.69–18.67)	15.71(14.23–16.43)	18.35(16.48–19.40)	0.001^*^
All-cause mortality,%	21.05(8/38)	25.00(5/20)	16.67(3/18)	0.277
All adverse effects [%, no.]	31.58(12/38)	15.00(3/20)	50.00(9/18)	0.022^*^
Nausea/Vomiting [%, no.]	10.53(4/38)	5.00(1/20)	16.67(3/18)	
Hyperkaliemia [%, no.]	7.89(3/38)	5.00(1/20)	11.11(2/18)	
Diarrhea [%, no.]	7.89(3/38)	0.00(0/20)	16.67(3/18)	
Thrombocytopenia [%, no.]	2.63(1/38)	5.00(1/20)	0.00(0/18)	
Drug eruption [%, no.]	2.63(1/38)	0.00(0/20)	5.56(1/18)	

ST group, synergic therapy group; MT group, monotherapy group;

### Changes in inflammatory biomarkers and ventilator parameters after GCS use

The ventilator parameters and inflammatory biomarkers were measured, and 15 patients in the ST group who were intubated were included in the analysis. There was no significant difference between the ventilator parameters before and after the use of the GCS. After the GCS was administered, the IL-6 level significantly decreased. However, there was no significant difference in CRP levels before or after the use of the GCS (Table 5).

**Table 5:** Change of inflammatory biomarkers and ventilator parameters after the utilization of GCS

Parameters	0 day of the utilization of GCS	3 days after the utilization of GCS	*P*
Interleukin-6, pg/mL, [median (IQR)]	268.00(23.74–482.90)	43.00(25.00–56.00)	0.040*
C-reactive protein, mg/L, [median (IQR)]	66.70(54.20–168.00)	67.00(43.00–90.00)	0.619
Ventilator parameters			
V_T_, ml/kg(PBW)[median (IQR)]	5.40(5.00-6.30)	5.40(5.20–6.10)	0.095
PEEP median, cmH_2_O [median (IQR)]	10.00(8.00–11.00)	8.00(8.00–10.00)	0.189
Plateau pressure, cmH_2_O median (IQR)]	22.00(20.00–28.00)	21.00(18.00–23.00)	0.196
Driving pressure, cmH_2_O [median (IQR)]	12.00(11.00–16.00)	12.00(9.00–15.00)	0.271

GCS, glucocorticosteroid; PEEP, Positive End Expiratory Pressure

### A typical case report from the ST Group

**Fig. 1:**
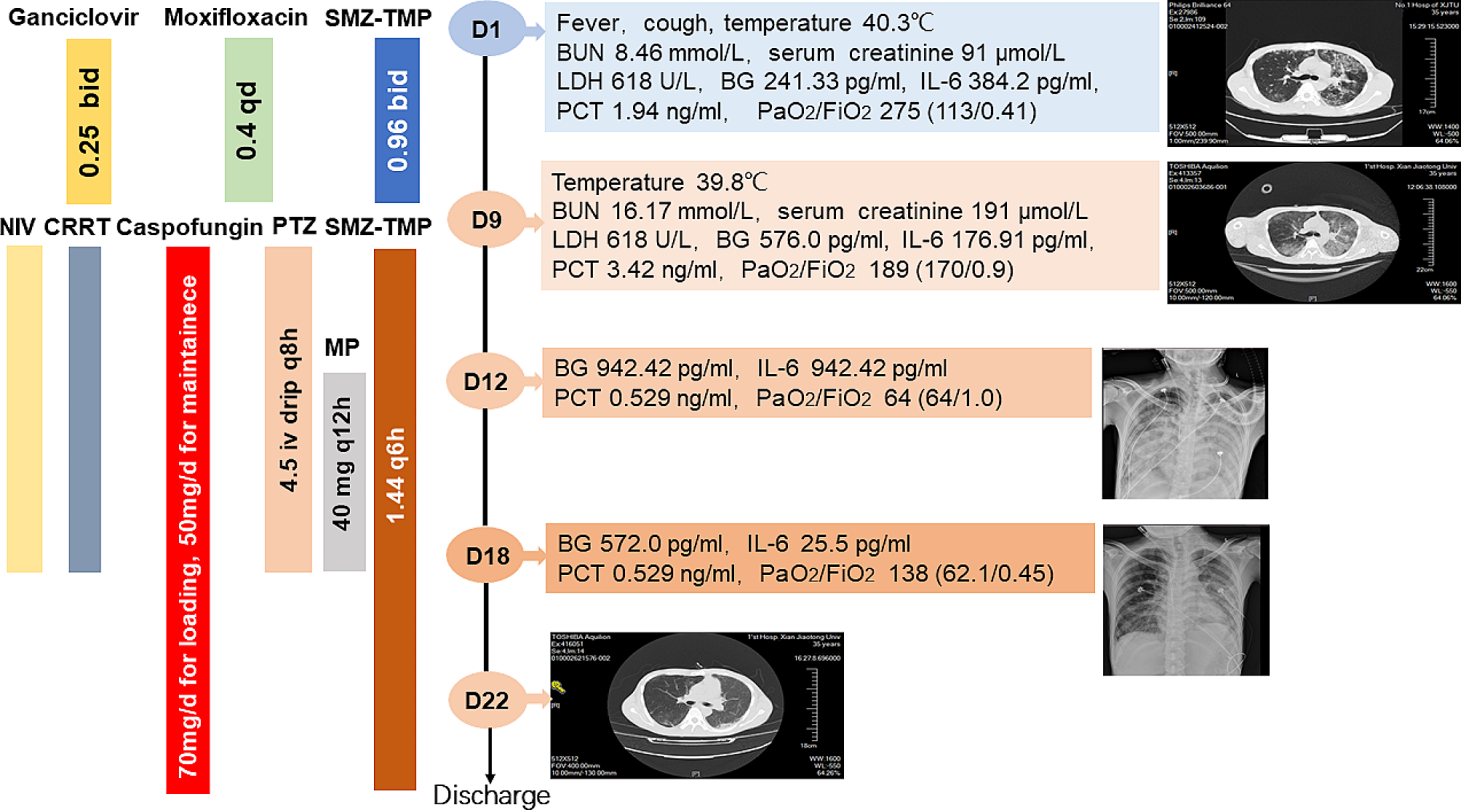
Timeline of the case. SMX-TMP, sulfamethoxazole-trimethoprim; BUN, blood urea nitrogen; LDH, lactate dehydrogenase; IL-6, interleukin-6; BG, β-(1,3)-D-glucan; PCT, procalcitonin; NIV, noninvasive ventilation; CRRT, continuous renal replacement therapy; PTZ, piperacillin-tazobactam; MP, methylprednisolone

**Fig. 2:**
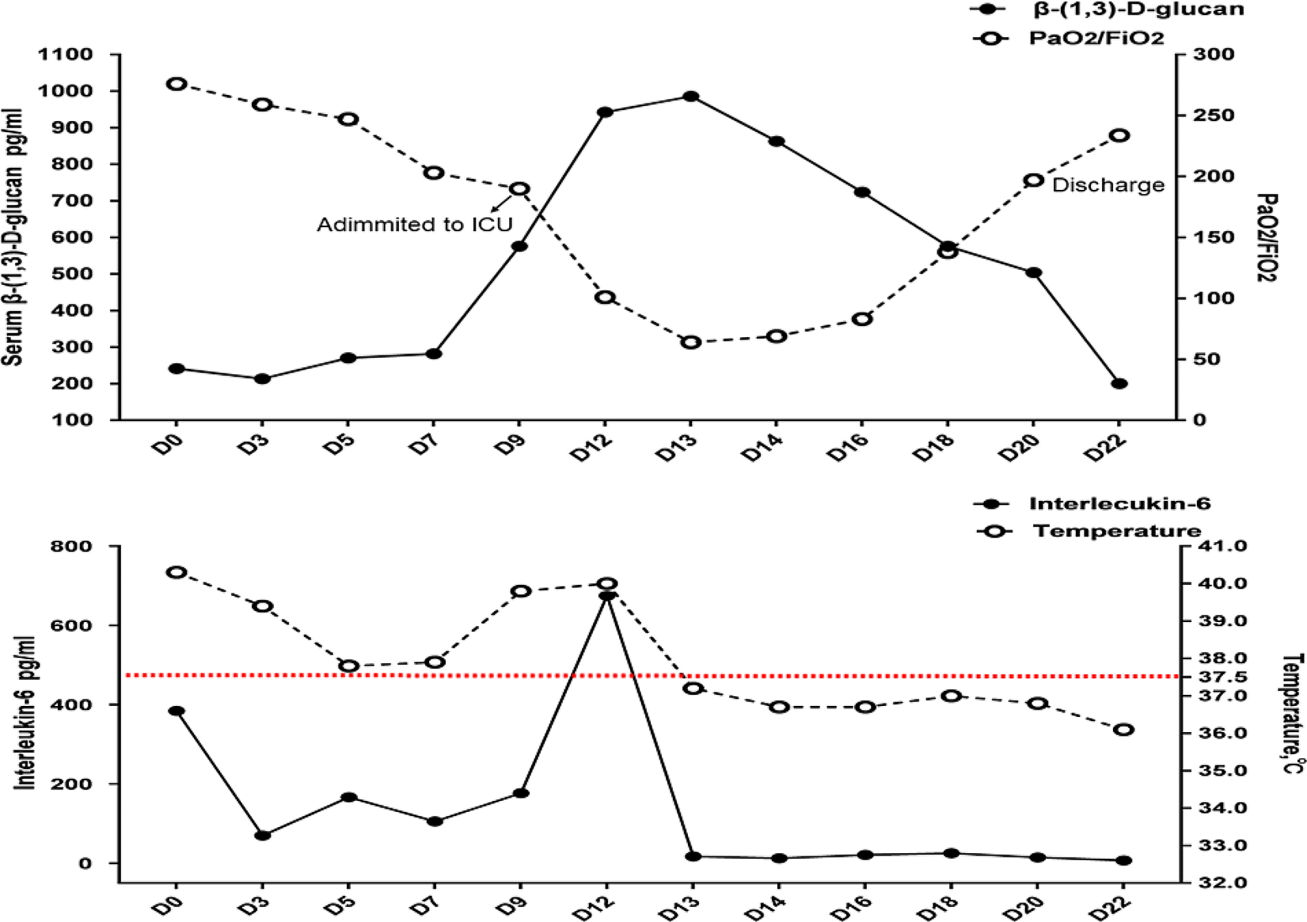
Trends in the serum β-(1,3)-D-glucan concentration, interleukin-6 concentration, PaO_2_/FiO_2_ ratio and temperature during the course of illness

A 34-year-old male patient who underwent renal transplantation 5 months before admission complained of a 4-day history of fever and cough. A lung computed tomography (CT) scan showed bilateral diffuse ground-glass opacities. The patient initially underwent oxygen therapy via nasal prongs. Moxifloxacin (400 mg/day) and ganciclovir (250 mg every 12 h) were administered as empirical anti-infective therapies, and SMZ-TMP (800/160 mg every 12 h) was used as prophylaxis for PJP. After 7 days, the patient’s symptoms did not improve, and he developed respiratory distress, hypoxemia, and oliguria. The lung CT scan showed massive area consolidation. The patient was subsequently transferred to the ICU for noninvasive ventilation, and BALF mNGS revealed *P. jirovecii* infection (read 18,117); his antibiotic treatment was subsequently changed to SMZ-TMP (1200/240 mg every 6 h, which equals 15 mg/kg TMP per day), caspofungin (70 mg as the loading dose and 50 mg/day as the maintenance dose) and piperacillin-tazobactam (4.5 g every 8 h). Because of the kidney injury and oliguria, the patient received continuous renal replacement therapy. However, after 2 days of therapy, the PaO_2_/FiO_2_ ratio decreased. Considering that the patient developed ARDS and severe PJP, methylprednisolone (40 mg bid) was added. The clinical condition of the patient improved, his PaO_2_ gradually increased to 109 mmHg (FiO_2_ 40%), and his temperature decreased to a normal level. On Day 22, the patient was discharged from the ICU without the need for noninvasive ventilation. A detailed description of this case is shown in Figs. 1 and 2.

## Discussion

This retrospective study aimed to evaluate the efficacy and safety of a regimen based on the combination of SMZ-TMP with caspofungin and GCSs for the treatment of non-HIV-related PJP. Ultimately, our results showed that synergistic therapy, as a first-line therapy for non-HIV-related PJP, can increase the clinical response rate, decrease the IL-6 level, shorten the duration of fever and decrease the incidence of adverse events. To the best of our knowledge, this is the first study on the efficacy and safety of synergistic therapy for severe non-HIV-related PJP treatment in China.

**Fig. 3:**
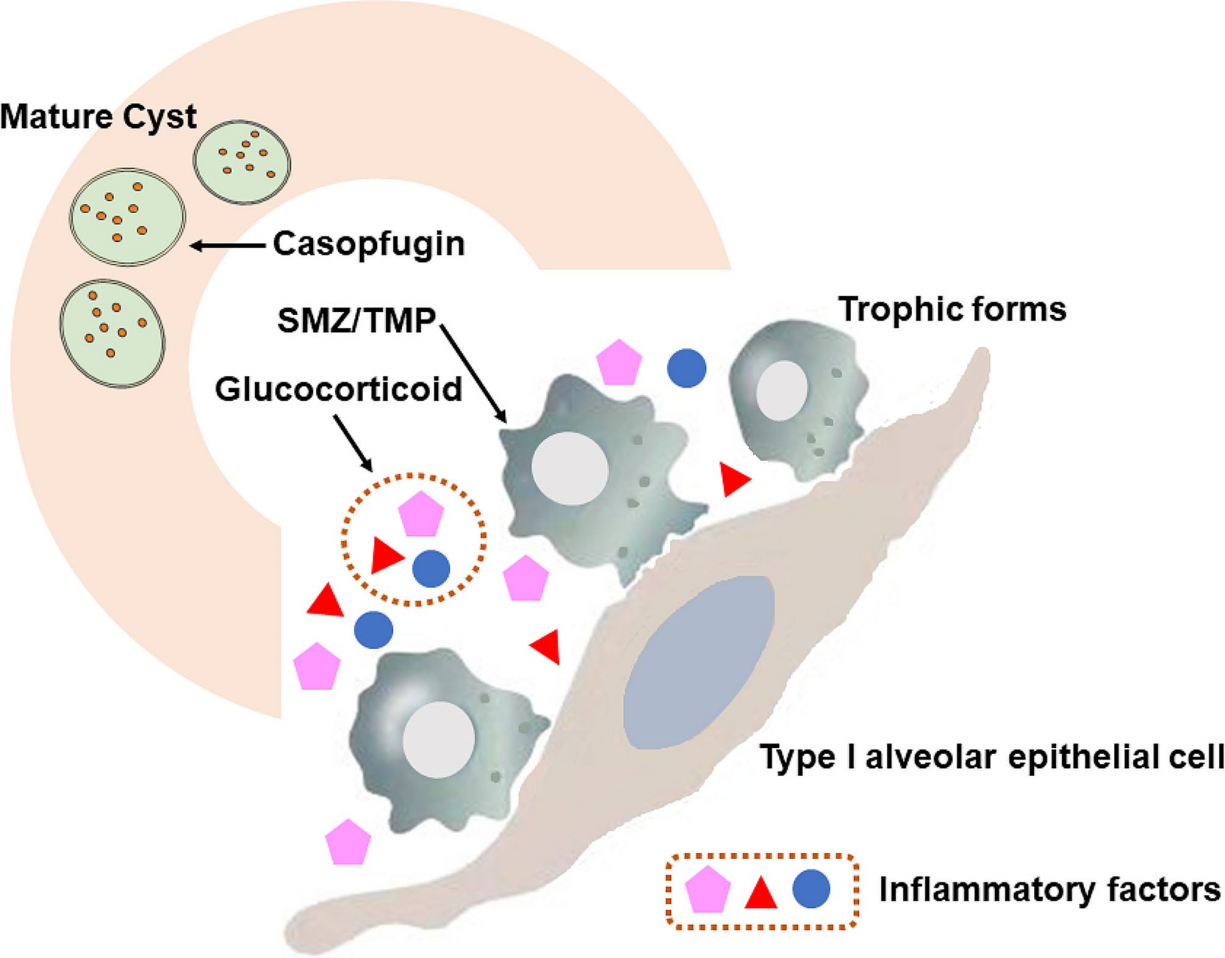
The life cycle of pneumocystis in the lung and the mechanism of synergistic therapy SMX-TMP, sulfamethoxazole-trimethoprim

Despite the use of SMZ-TMP, the mortality of non-HIV-related PJP remains high, the treatment of PJP is still a major challenge for clinical physicians, and alternative therapies need to be explored. Furthermore, in our hospital and most of China, pentamidine, primaquine and atovaquone are unavailable, which means that for patients receiving SMZ-TMP with treatment failure or intolerance, the choice of salvage therapy is very limited. *Pneumocystis jirovecii* is a pathogen with a biphasic life cycle in the host lungs that consists of trophic forms and mature cysts [6]. The trophic forms of *Pneumocystis jirovecii* are the most abundant forms, and the life cycle can be initiated by adhering to type I alveolar epithelial cells during initial infection [6, 19, 20]. On the other hand, the cell walls of mature cysts contain β-(1,3)-D-glucan, which can stimulate alveolar macrophages to release inflammatory factors, such as TNF-α, and ultimately cause inflammatory responses in the lungs [2, 20]. In addition, some studies have demonstrated that the adherence of trophic forms does not disrupt the structure of alveolar cells, which means that the exuberant inflammatory host response is the main factor responsible for lung injury and respiratory distress in PJP patients [21, 22]. In summary, three key components are involved in the pathogenesis of PJP: (1) The trophic forms of *Pneumocystis jirovecii* adhere to alveolar epithelial cells. (2) The β-(1,3)-D-glucan in mature cysts stimulates the inflammatory response in the host. (3) The inflammatory response injures the lung and induces ARDS. Therefore, PJP should be treated directly through these three key steps, and synergistic therapy with multiple drugs may be more effective than SMZ/TMP monotherapy. As shown in Fig. 3, in the present study, we used the combination of SMX-TMP with caspofungin and a GCS as a synergistic therapy. SMZ/TMP is used against trophic forms, caspofungin kills mature cysts, and GCS inhibits the inflammatory response and prevents lung injury. As expected, compared with those in the MT group, the clinical positive response was significantly greater, the incidence of adverse events was lower, and the duration of fever was shorter in the ST group. These findings indicate that the combination of SMX-TMP with caspofungin and a GCS can block the pathogenesis of different types of PJP and improve symptoms, which may be more effective and safer than SMZ/TMP monotherapy is (Table 4).

In the present study, caspofungin was used in combination with SMZ/TMP to treat severe non-HIV-related PJP. However, studies on caspofungin for the treatment of non-HIV-related PJP are limited, and most previous studies are case reports. In 2007, Utili R et al. published the case reports of 4 non-HIV-related PJP patients with severe hypoxemia who were treated with a combination of SMZ/TMP and caspofungin, and the combination regimen led to rapid improvement of infection and a good clinical outcome [5]. Tu et al. also described the cases of 3 renal transplantation patients with severe PJP who received treatment with a combination of SMZ/TMP and caspofungin and showed a good response with a reduction in the incidence of SMZ/TMP adverse effects [1]. On the other hand, Lobo ML et al. reported that the combination of SMZ/TMP and caspofungin resulted in rapid clearance of Pneumocystis infection in an immunosuppressed mouse model, and the combination regimen was more effective than either TMP-SMZ alone or caspofungin alone [6]. In 2018, Zhang et al. indicated that the combination of SMZ/TMP and caspofungin may be an effective strategy for treating non-HIV patients with PJP, especially for patients requiring mechanical ventilation [8]. Based on these studies, the Pneumocystis jirovecii in solid organ transplantation: Guidelines from the American Society of Transplantation Infections Diseases Community of Practice recommend the combination of SMZ/TMP and caspofungin as an option for treatment (low-level evidence) [23]. Furthermore, the ECIL guidelines recommend the combination of SMZ/TMP and caspofungin as a second-line regimen for PJP in patients with haematological malignancies, solid organ transplants or cancer (low-level evidence) [4]. However, clinical trials comparing the efficacy of SMZ-TMP plus caspofungin versus SMZ-TMP alone are very limited. In the future, clinical trials that compare the efficacy of SMZ-TMP plus caspofungin versus SMZ-TMP alone for the treatment of severe non-HIV-related PJP should be performed.

The benefit of utilizing GCSs for the treatment of non-HIV-related PJP has not been validated.

The Pneumocystis jirovecii in solid organ transplantation: Guidelines from the American Society of Transplantation Infections Diseases Community of Practice indicate that.

In patients with hypoxemia, the use of adjunctive GCSs remains controversial but is recommended regardless of the antimicrobial agent selected, and GCS therapy should be considered early for maximum benefit [23]. As shown in the present study, we found that the use of a GCS can significantly decrease the level of IL-6 (Table 5), which indicated that the use of a GCS can decrease the inflammatory response in non-HIV-related PJP patients. However, GCS use had no effect on the CRP level. This discrepancy may be caused by several factors. First, there are multiple factors, including inflammation, infection, thromboembolism, cardiovascular disease and tissue damage, that may affect CRP levels. Second, in comparison with IL-6, CRP is a less reliable and sensitive biomarker [24].

On the other hand, there are limited data about the use of GCSs for non-HIV-related PJP patients. Pareja et al. indicated that in patients with severe non-HIV-related PJP, a dose of more than 60 mg prednisone per day (methylprednisolone 48 mg per day) may accelerate recovery and decrease the duration of mechanical ventilation and the duration of hospital stay [3]. On the other hand, Lemiale et al. included 139 non-HIV-related PJP patients and demonstrated that a high dose of a GCS (≥ 1 mg/kg/day prednisone equivalent) was associated with increased mortality via a mechanism independent of an increased risk of infection [25]. The duration and dosage of the GCS were not indicated in the studies of both Pareja et al. and Lemiale et al. According to the case report in the present study, we hope to explain the reason for the use of the GCS and the time to cessation of treatment. At the diagnosis of PJP on Day 9, SMX-TMP combined with caspofungin was administered. However, after therapy, the PaO_2_/FiO_2_ ratio decreased, the chest X-ray results worsened, the IL-6 and β-(1,3)-D-glucan levels increased markedly, and the patient developed severe ARDS. We speculated that the worsening of the patient’s condition could be related to the inflammatory response. After the use of SMZ/TMP and caspofungin, the broken mature cysts may have released large amounts of β-(1,3)-D-glucan, which can induce the release of inflammatory factors and lung injury. In addition, the mean dosage and duration of methylprednisolone were 0.96 ± 0.46 mg/kg/day and 6.71 ± 2.57 days, respectively, in the present study; these values were less than 1.0 mg/kg/day (a high dosage of methylprednisolone), and the duration of GCS use was short. Therefore, we speculated that a short duration of low-dose GCS use may be helpful for inhibiting the inflammatory response and improving lung injury. On the other hand, we used the kinetics of β-(1,3)-D-glucan to determine the appropriate time to cease GCS use. As shown in Fig. 2, the β-(1,3)-D-glucan concentration peaked on Day 13, and after the use of GCS, the β-(1,3)-D-glucan concentration decreased gradually. On Day 22, the β-(1,3)-D-glucan level returned to the baseline level. We considered that the reduction in β-(1,3)-D-glucan could indicate a decrease in β-(1,3)-D-glucan-induced inflammation; subsequently, we ceased GCS use. In addition, another reason we were motivated to stop the GCS was the improvement in the PaO_2_/FiO_2_ ratio on Day 18. Therefore, based on our clinical experience and the present study, we speculated that (1) it may be helpful to use GCSs at a low dose and for a short duration of treatment for severe non-HIV-related PJP. (2) Increases in IL-6 and β-(1,3)-D-glucan levels and a reduction in the PaO_2_/FiO_2_ ratio may indicate the need to start using a GCS, and the kinetics of β-(1,3)-D-glucan and the improvement in PaO_2_/FiO_2_ ratio may be used to determine the time at which to discontinue GCS use. (3) The risk of infection should be considered after the use of a GCS. (4) The duration of utilization of GCSs should be short. However, since non-HIV-related PJP patients are mainly immunocompromised, it is difficult to determine the main risk factor for infection: the host response or the GCS? Since the present study involved a small sample size, further studies should be performed to explore the duration and dosage of GCSs for the treatment of severe non-HIV-related PJP, and both the anti-inflammatory response and decreased infection should be considered.

According to our results, the incidence of adverse effects in the ST group was lower than that in the MT group (Table 4). This difference may have been caused by the lower dose of SMZ/TMP in the ST group. We speculate that this difference may have been due to the better effect of synergistic therapy, and there was no need to use higher doses of SMZ/TMP in the ST group.

There are several limitations in our study: (i) This present study had a small sample size and retrospective design, which may have resulted in bias and a false-positive (type I) error. In the future, a prospective, controlled, large sample clinical trial of severe non-HIV-related PJP patients is needed to determine the benefit of synergistic therapy and SMZ/TMP monotherapy. (ii) The population in the present study mainly included patients with severe non-HIV-related PJP, and we do not know whether synergistic therapy is appropriate for patients with mild-moderate non-HIV-related PJP. (iii) It was difficult to determine the benefit of the GCS for the treatment of severe non-HIV-related PJP in the present study. In the future, clinical trials that compare the efficacy of SMZ-TMP plus a GCS versus SMZ-TMP alone for the treatment of severe non-HIV-related PJP should be performed. However, the duration and dosage of GCSs for the treatment of severe non-HIV-related PJP have not been determined.

In conclusion, the present study showed that, in comparison with SMZ/TMP monotherapy, synergistic therapy (SMZ-TMP combined with caspofungin and a GCS) to treat severe non-HIV-related PJP can increase the clinical response rate and decrease the incidence of adverse events. Synergistic therapy may be effective and safe as a first-line therapy for treating severe non-HIV-related PJP.

## Data Availability

The datasets generated during and/or analysed during the current study are not publicly available for confidentiality reasons but are available from the corresponding author upon reasonable request.
